# Neurological symptoms in a case of acute aortic dissection

**DOI:** 10.1590/0100-3984.2015.0070

**Published:** 2016

**Authors:** Igor Aloísio Garcez Zamilute, Fabiano Reis, Nivaldo Adolfo Silva Junior, Tania Aparecida Marchiori de Oliveira Cardoso, Wendy Caroline de Souza Costa França

**Affiliations:** 1 Faculdade de Ciências Médicas da Universidade Estadual de Campinas (FCM-Unicamp), Campinas, SP, Brazil.

**Figure 1:**
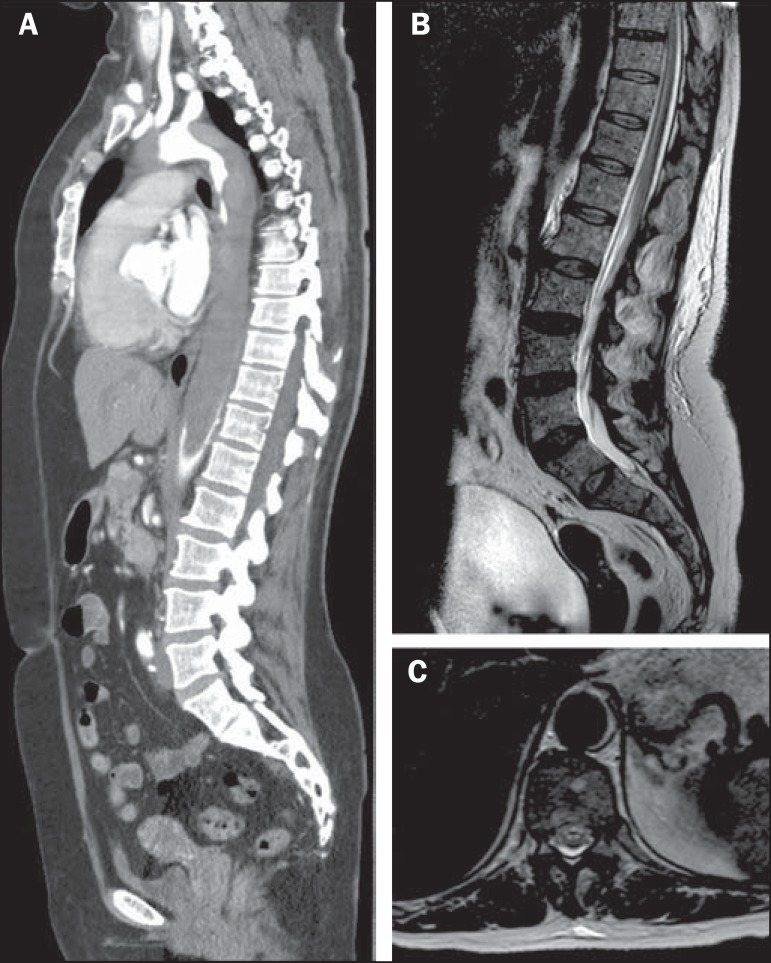
**A:** Maximum intensity projection reconstruction of computed tomography angiography in the sagittal plane showing an extensive mural thrombus in the thoracic aorta, extending to the infrarenal aorta. **B:** Sagittal T2-weighted MRI sequence showing areas of hyperintensity within the anterior spinal cord. **C:** Axial T2-weighted MRI at the level of the T10 spinal segment demonstrating anterior areas of hyperintensity, with the "owl eye" sign.

*Dear Editor*,

A 52-year-old female with aortic dissection presented with neurological symptoms and signs, in a markedly acute presentation, of flaccid paraplegia and painful hypoesthesia of the lower limbs. She also presented postoperative monoplegia of the left arm. Computed tomography angiography of the chest confirmed the diagnosis of type A dissection (Stanford classification), with extension to the infrarenal abdominal aorta, associated with extensive subocclusive thrombus in the thoracoabdominal transition of the aorta (Figure 1A). On T2-weighted magnetic resonance imaging (MRI) sequences, hyperintensity was observed in the anterior horns of the spinal cord (Figures 1 B and 1C), featuring an "owl eye" sign in axial images^(1)^, together with enhancement after administration of paramagnetic contrast, as well as restricted diffusion of water at the levels studied. Cranial MRI revealed acute lesions (also with restricted diffusion) in the right middle cerebral artery. The patient underwent surgery to treat the aortic dissection, and her neurological function was monitored.

The evaluation of the aorta by imaging methods has been the subject of a series of recent publications in the Brazilian radiology literature^(2-4)^. In the case presented here, neurological findings were associated with aortic dissection, and the MRI findings were consistent with the diagnosis of spinal cord infarction with ischemic stroke in the right middle cerebral artery. Although spinal cord infarction is not a rare event^(5)^, the subtlety of the findings and the wide range of differential diagnoses can make its diagnosis difficult. Spinal cord ischemia can be attributed to several causes, including aortic dissection (as in the case presented) and thoracolumbar sympathectomy, or can even occur as a postpartum complication. The single anastomotic segment that irrigates the anterior two thirds of the spinal cord (mainly by the artery of Adamkiewicz) is more susceptible to ischemia than is the posterior segment, which has several levels of vascular supply^(6)^. A high degree of clinical suspicion of neurological involvement of the spinal cord is indicative of the diagnosis. Symptoms vary depending on the extent of the affected area and the level of spinal injury. Cerebral ischemic lesion is also a possible complication of aortic dissection and can result from reduced blood flow to the brain caused by the surgical procedure or even from carotid involvement caused by dissection or embolism from the thrombus in the aorta. In addition, data in the literature indicate that there is a right-side dominance of lesions, which is explained by different mechanical dynamics in the progression of the dissecting hematoma.

MRI is particularly sensitive in the detection of aortic dissection and can reveal signal abnormality in the anterior horns of the spinal cord, which can be associated with enhancement after contrast agent injection. The spinal segment most often affected is the thoracic segment, due to the border arterial supply^(6)^. Diffusion sequences can show restriction in the ischemic area. In fact, diffusion sequences can provide early detection^(7)^, although this technique is not always applied in routine MRI scans of the spinal cord. Therefore, we have presented a case of aortic dissection with a rare combination of neurological complications of brain and spinal cord ischemia.
